# Transcriptome analysis of ageing in a long-lived seabird reveals sex-specific differences across age classes in immune and DNA repair pathways

**DOI:** 10.3389/fragi.2026.1880546

**Published:** 2026-07-15

**Authors:** Beatrice Berardi, Giacomo Dell'Omo, David Costantini

**Affiliations:** 1 Department of Biology and Ecology, University of Tuscia, Viterbo, Italy; 2 Ornis Italica, Rome, Italy

**Keywords:** aging, gene expression, longevity, RNA-seq, Scopoli’s shearwater, sex-differences, transcriptome

## Abstract

**Introduction:**

Sex differences in ageing rate and longevity are widespread across animals, but their molecular bases remain poorly understood. Ageing involves multiple hallmarks of functional decline, and experimental studies suggest sex-specific differences in gene expression, particularly in immune, metabolic, and mitochondrial pathways. Yet, transcriptional variation associated with sex and age in natural populations remains largely unexplored.

**Methods:**

Here, we present the first RNA-seq study of a free-living bird, the Scopoli’s shearwater (*Calonectris diomedea*), aimed at investigating sex differences in gene expression across age classes, comparing younger and older individuals of both sexes (16 males and 16 females).

**Results:**

We identified a sex-linked transcriptional signature involving genes associated with immune function, stress response, energetics, and development. Age-related reductions in energy metabolism were more pronounced in females, whereas the strongest sex-related differences were detected in immune function and genome stability pathways. Across age classes, males showed higher expression of genes involved in DNA repair and genome maintenance, while females displayed a shift from immune-related processes in early adulthood to stress-response pathways in late adulthood.

**Discussion:**

Together, these findings reveal pronounced sex-biased transcriptional regulation across age classes, which we hypothesise is driven by the differentiation of sex-specific reproductive strategies during the egg-laying phase. Furthermore, our results indicate sustained investment in somatic maintenance into late adulthood, consistent with patterns typically observed in long-lived species.

## 1 Introduction

There is substantial variation in ageing rates and lifespan between females and males across animal species. In mammals, females generally live longer, age more slowly, and cease reproduction earlier than males, whereas the opposite pattern is often observed in birds (Austad and Fischer, 2016; Clutton-Brock and Isvaran, 2007; Lemaître et al., 2020; Staerk et al., 2025). Three main, non-mutually exclusive hypotheses have been proposed to explain sex differences in ageing and lifespan. First, the “adaptive sexual selection hypothesis” argues that sex-specific selection pressures shape life-history strategies, with the more competitive sex (typically males) investing more in reproduction at the expense of longevity (Maklakov and Lummaa, 2013). Second, the “unguarded X hypothesis” suggests that the heterogametic sex (e.g., mammalian males, avian females) tends to have a shorter lifespan because having only one copy of the sex chromosome allows deleterious recessive mutations to remain unmasked and be expressed (Trivers, 1985). Finally, the “mother’s curse hypothesis” highlights the role of maternal mitochondrial inheritance, whereby mtDNA mutations that are harmful to males but neutral in females can escape purifying selection, accumulate over time, and contribute to reduced male fitness and longevity (Frank and Hurst, 1996). Empirical support for these hypotheses in natural animal populations is largely limited: the sexual selection hypothesis was mainly tested in polygamous systems (Edmands, 2024), whereas evidence in support of the unguarded X or the mother’s curse hypotheses comes mostly from studies on mammals and *Drosophila* fruitflies (Cayuela et al., 2023; Connallon et al., 2022).

Most studies addressing sex-specific biological variation focus primarily on phenotypic or strictly genetic components of ageing-related processes. There is now growing consensus that ageing is driven and characterized by a series of hallmarks that encompass a wide range of molecular, cellular, and systemic mechanisms, which together contribute to increasing genomic instability with advancing age (López-Otín et al., 2023). Therefore, identifying the proximate mechanisms underlying sex-biased molecular regulation over the lifespan remains a key unresolved question, and is essential for understanding the sources of variation in ageing in the wild. For example, prior studies have suggested that potential mechanisms underlying sex-biased differences in ageing may lie in the endocrine system (Garratt and Stout, 2018; Adámková et al., 2019), resistance to oxidative stress to oxidative stress (Costantini, 2024) or immune function (Metcalf et al., 2020). Pathways related to stress responses, hormone synthesis, inflammation, and immunity are also relevant mechanisms linked to ageing across multiple species (Alonso-Alvarez et al., 2010; Lin et al., 2017; Těšický et al., 2021; Berardi et al., 2026a). However, empirical evidence is not always consistent across studies, as young and old individuals of both sexes may not differ in immune function, oxidative status, or other physiological parameters (Montgomery et al., 2012; Bichet et al., 2022).

The absence of detectable phenotypic differences does not preclude the occurrence of age- and sex-related molecular changes (e.g., gene expression), which may remain cryptic when assessed using conventional physiological endpoints. Recent studies found pronounced sex differences in age-associated gene expression in humans and mice, particularly in pathways related to immune signalling and response, as well as amino acid, lipid, and mitochondrial energy metabolism (Achiro et al., 2024; Marttila et al., 2013; Zhu et al., 2024). However, sex-biased transcriptional regulation across lifespan has not yet been investigated in natural populations of mammals or birds.

To the best of our knowledge, we present the first transcriptomic study in a free-living bird aimed at identifying candidate genes and pathways underlying sex differences across age classes. Specifically, we performed RNA-seq on the blood transcriptome of adult Scopoli’s shearwaters (*Calonectris diomedea*) of both sexes, comparing (i) younger versus older females, (ii) younger versus older males, (iii) younger females versus younger males, and (iv) older females versus older males.

The Scopoli’s shearwater is a long-lived, monogamous seabird that can live beyond 30 years. Previous studies on other Procellariiform species have reported sex-specific patterns of ageing, particularly in survival, breeding success, and endocrine regulation, with males often showing stronger signs of ageing than females (Angelier et al., 2006; Pardo et al., 2013). Based on these findings, we expected the shearwater transcriptome to exhibit sex-biased transcriptional patterns across age classes, particularly in immune, metabolic, and hormonal pathways, which are central to trade-offs between somatic maintenance and reproduction.

## 2 Materials and methods

### 2.1 Study area and sample collection

The individuals of Scopoli’s shearwaters used for this study breed on Linosa Island (Sicily Channel, south-western Mediterranean Sea; 35°52′ N, 12°52′ E). The birds nest in ground-level cavities in the lava rock, which are readily accessible and allow individuals to be gently captured by hand. The species is monogamous, and both parents participate in incubation and chick rearing. Breeding occurs annually, with each pair laying a single egg in May that is incubated for approximately 52 days. Consequently, all sampled individuals were engaged in their first and only reproductive attempt of the season. We collected samples in May 2024. Birds (16 males and 16 females) were sampled after incubation had begun. Breeding status was confirmed by verifying the presence of an egg in the nest, whereas age was determined based on each individual’s ringing history. Individuals aged 5–9 years (n = 16) were classified as younger adults, as Scopoli’s shearwaters typically begin breeding at approximately 5–6 years of age. Based on previous analyses conducted on a large historical dataset from the colony, we know that early adulthood (i.e., the juvenile-to-adult transition phase during which individuals reach full physiological maturity and acquire the experience needed to successfully raise a chick) spans approximately 5–9 years of age (Berardi et al., 2026a). The older adults (n = 16) encompassed a broad age range, as few individuals survive beyond 19 years of age, leading to a sparse and discontinuous representation of older age classes. Therefore, in this group only two individuals (one male and one female) reached 36 years of age, while the remaining were between 19 and 22 years old (Supplementary Table S1 reports the distribution by sex and age class). Consequently, age classes should be considered as a simplified, two-category grouping designed approximate and compare broad life stages, namely, earlier versus later phases of the life cycle. Sex was determined based on the marked sexual dimorphism in adult body mass, particularly evident during the egg-laying period, with males being heavier than females, as well as on beak size, vocalizations, and repeated measurements collected from both members of each breeding pair over multiple years (Massa and Lo Valvo, 1986; Becciu et al., 2012).

From each individual, we collected 400 µL of whole blood from the tarsal vein within 5 min of capture using a 1 mL syringe. Straightaway the collection of blood, we stabilised it in RNAprotect Animal Blood Tubes (QIAGEN Inc.) to prevent RNA degradation. Afterwards, we stored the samples at −20 °C in the field facilities and at −80 °C when back to the laboratory until analyses.

### 2.2 RNA extraction and library construction

After extraction and purification of RNA, we performed the quality check using the Agilent 5,400 Fragment Analyzer (Agilent Technologies, CA, United States). All samples passed the quality check and were therefore used for library construction. Messenger RNA was purified from total RNA using poly-T oligo-attached magnetic beads. After fragmentation, the first strand cDNA was synthesized using random hexamer primers, followed by the second strand cDNA synthesis using either dTTP for non-strand specific library or dUTP for strand specific library. The library was checked with Qubit and real-time PCR for quantification and bioanalyzed for size distribution detection. Quantified libraries were sequenced on Illumina platforms at Novogene.

### 2.3 Data quality control and filtering

Filtering of raw reads was done as follows. First, we removed reads containing adapters. Then, we removed reads if more than 10% of the total bases in a read were undetermined. Finally, we discarded further reads if more than 50% of the bases had a Q-score ≤5. We used HISAT2 v.2.0.5 to map the clean reads to the reference genome of *Calonectris borealis* (Cory’s shearwater) (https://www.ncbi.nlm.nih.gov/datasets/genome/GCF_964195595.1/). The genome sequence records for Calonectris borealis RefSeq assembly GCF_964195595.1 (bCalBor7.hap1.2) were annotated by the NCBI Eukaryotic Genome Annotation Pipeline, an automated pipeline that annotates genes, transcripts and proteins on draft and finished genome assemblies.

Genes with a total read count (rowSums) greater than 1 across all samples were retained for differential expression analysis. We calculated FPKM values using the fpkm() function in the DESeq2 R package, which computes gene length-normalized expression values directly from a DESeqDataSet object.

### 2.4 Statistical analyses

We quantified the differences in blood transcriptome between (i) younger females and older females, (ii) younger males and older males, (iii) younger females and younger males, and (iv) older females and older males. We performed the differential expression analysis using models based on negative binomial distribution in the DESeq2 R package following Love et al. (2014). The resulting p-value was adjusted using the Benjamini and Hochberg’s method to control the error discovery rate (Green and Diggle, 2007). Statistical significance was determined using the Wald test, and the threshold of significant differential expression was p-value adjusted ≤0.05 and |log2(foldchange)| ≥ 1. FPKM values were used only for visualization and descriptive analyses (e.g., heatmaps and expression plots). Then, we implemented Gene Ontology (GO) enrichment analysis of differentially expressed genes by the cluster Profiler R package, in which gene length bias was corrected (Young et al., 2010). Finally, we used the database KEGG for identifying high-level functions and utilities of biological systems, and the KEGG pathway enrichment to identify the most significant pathways that differ the most between groups (Kanehisa and Goto, 2000; Kanehisa, 2002). Enriched GO terms and KEGG pathways with an adjusted p-value <0.05 were considered statistically significant.

Given the high number of differentially expressed genes found between younger females and younger males, as well as between older females and older males, we performed an additional analysis using the R package *dplyr* (Wickham et al., 2026; version 1.2.0) to identify genes that are constitutively differentially expressed between sexes throughout life from those whose differential expression is age-dependent. Afterwards, to facilitate visualization and interpretation of these results, genes were grouped into functional categories (immune, mitochondrial, stress response, DNA repair, and others) based on their annotated functions to enable bar chart construction (See Results, Section 3.5). Gene assignment was performed in R using the packages *dplyr* and *tidyr* through keyword-based searches of functional annotations.

## 3 Results

High-throughput RNA sequencing generated a total of 2,288,091,994 raw paired reads, ranging from 59,453,296 to 96,326,112 per individual (mean: 71,502,874.81), and a total of 2,267,772,260 clean reads ranging from 58,983,350 to 95,052,084 per individual. The percentage of reads being mapped to unique positions of the reference genome (for subsequent quantitative data analysis) ranged between 77.46% and 90.83% per individual, a much higher percentage than the 60% basic requirement for non-model organisms.

### 3.1 Younger females versus older females

**FIGURE 1:**
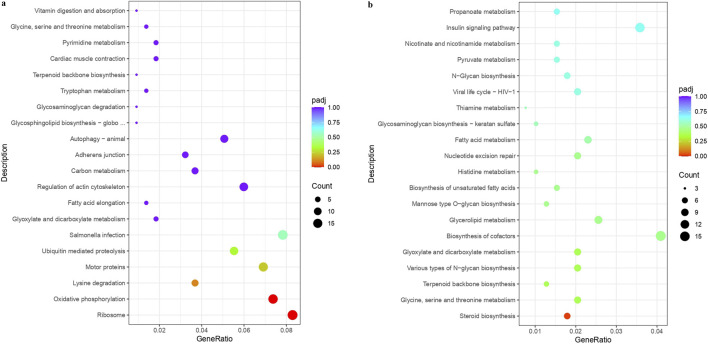
KEGG enrichment analyses of differential expressed genes between **(a)** younger and older females, and **(b)** younger females and younger males. The size of each point represents the number of genes associated with the corresponding term, as indicated in the legend. The Gene ratio refers to the ratio of differentially expressed genes over all genes of a specific KEGG pathway. The adjusted *p*-value decreases from top to bottom.

**FIGURE 2:**
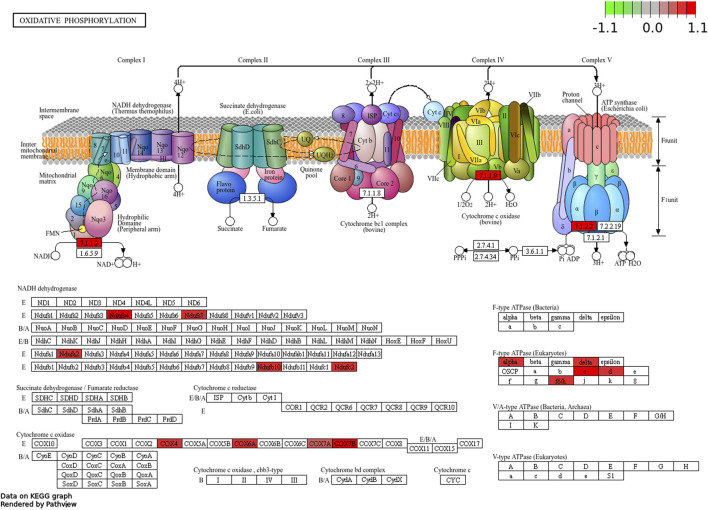
Visualization of the KEGG pathway for oxidative phosphorylation. Differentially expressed genes (log2 fold change) between younger and older females for each complex of the electron transport chain are highlighted in red rectangles. Red colour indicates higher expression in younger than in older females.

**FIGURE 3:**
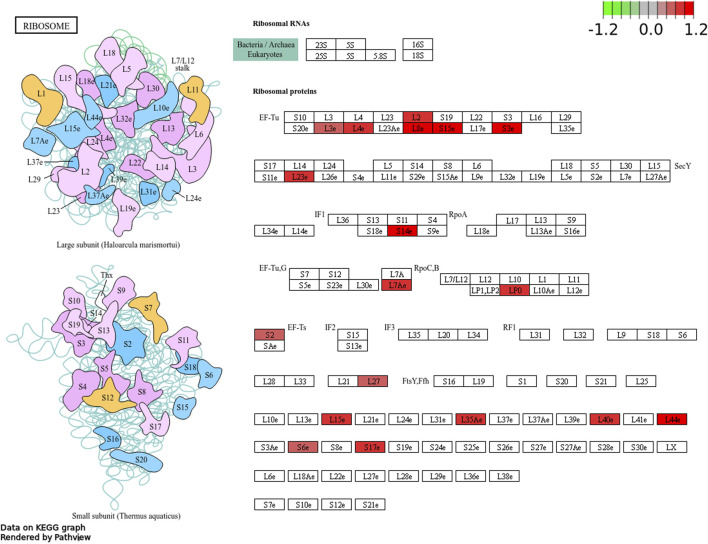
Visualization of the KEGG pathway for ribosome biogenesis. Differentially expressed genes (log2 fold change) between younger and older females for each ribosomal protein are highlighted in red rectangles. Red colour indicates higher expression in younger than in older females.

In the comparison between younger and older females, we identified three differentially expressed genes (Supplementary Table S2): uncharacterized LOC142075195, NT5DC3 (5′-nucleotidase domain containing protein 3), and MMTAG2 (Chromosome 1 Open Reading Frame 35). The log2 fold change values indicate that NT5DC3 is significantly downregulated in younger females compared to older females (log2FC = −3.88, Adj-p = 0.03). In contrast, MMTAG2 is significantly upregulated in younger females than in older females (log2FC = 1.30, Adj-p = 0.03). Gene Ontology enrichment analysis identified 42 significantly enriched terms (Table 1), which involve genes linked to mitochondrial activity, ATP energy metabolism, protein translation, and ribosome biogenesis. KEGG enrichment analysis revealed two significantly enriched pathways: ribosome and oxidative phosphorylation (Figure 1a). Younger females showed upregulation of genes involved in ribosome biogenesis and oxidative phosphorylation. In particular, 18 upregulated genes were associated with oxidative phosphorylation, including genes encoding components of the NADH dehydrogenase complex, cytochrome c oxidase, and the α and δ subunits of ATP synthase (Figure 2). Likewise, 18 upregulated genes were associated with ribosome biogenesis and encoded proteins of both the large and small ribosomal subunits (Figure 3).

**TABLE 1:** Gene Ontology (GO) classification of differentially expressed genes in adult Scopoli’s shearwaters, up- or down-regulated in younger compared with older females. The gene ratio refers to the proportion of genes associated with a given function relative to the total number of genes in the input list.

Category	GOID	Description	Gene ratio	P-adj	Count	Down	Up
BP	GO:0006412	Translation	26/176	3,70E+08	26	0	26
BP	GO:0043043	Peptide biosynthetic process	26/176	3,70E+08	26	0	26
BP	GO:0043604	Amide biosynthetic process	26/176	3,70E+08	26	0	26
BP	GO:0006518	Peptide metabolic process	26/176	5,54E+08	26	0	26
BP	GO:0043603	Cellular amide metabolic process	26/176	1,07E+09	26	0	26
BP	GO:1901566	Organonitrogen compound biosynthetic process	34/176	1,07E+09	34	3	31
BP	GO:0046034	ATP metabolic process	8/176	0,001	8	0	8
BP	GO:0009144	Purine nucleoside triphosphate metabolic process	8/176	0,001	8	0	8
BP	GO:0009199	Ribonucleoside triphosphate metabolic process	8/176	0,001	8	0	8
BP	GO:0009205	Purine ribonucleoside triphosphate metabolic process	8/176	0,001	8	0	8
BP	GO:0009141	Nucleoside triphosphate metabolic process	8/176	0,002	8	0	8
BP	GO:0009123	Nucleoside monophosphate metabolic process	8/176	0,003	8	0	8
BP	GO:0009126	Purine nucleoside monophosphate metabolic process	8/176	0,003	8	0	8
BP	GO:0009161	Ribonucleoside monophosphate metabolic process	8/176	0,003	8	0	8
BP	GO:0009167	Purine ribonucleoside monophosphate metabolic process	8/176	0,003	8	0	8
BP	GO:0017144	Drug metabolic process	8/176	0,009	8	0	8
BP	GO:0055114	Oxidation-reduction process	5/176	0,013	5	0	5
BP	GO:0015980	Energy derivation by oxidation of organic compounds	4/176	0,020	4	0	4
BP	GO:0009150	Purine ribonucleotide metabolic process	8/176	0,022	8	0	8
BP	GO:0009259	Ribonucleotide metabolic process	8/176	0,022	8	0	8
BP	GO:0019693	Ribose phosphate metabolic process	8/176	0,024	8	0	8
BP	GO:0006091	Generation of precursor metabolites and energy	5/176	0,024	5	0	5
BP	GO:1902600	Proton transmembrane transport	7/176	0,024	7	0	7
BP	GO:0006163	Purine nucleotide metabolic process	8/176	0,028	8	0	8
BP	GO:0006357	Regulation of transcription by RNA polymerase II	5/176	0,028	5	1	4
BP	GO:0072521	Purine-containing compound metabolic process	8/176	0,034	8	0	8
CC	GO:0005840	Ribosome	25/112	9,79E+04	25	0	25
CC	GO:0032991	Protein-containing complex	57/112	1,15E+05	57	5	52
CC	GO:1990904	Ribonucleoprotein complex	26/112	9,11E+05	26	0	26
CC	GO:0043228	Non-membrane-bounded organelle	38/112	1,42E+07	38	5	33
CC	GO:0043232	Intracellular non-membrane-bounded organelle	38/112	1,42E+07	38	5	33
CC	GO:0005737	Cytoplasm	43/112	2,38E+08	43	2	41
CC	GO:0005739	Mitochondrion	11/112	0,003	11	0	11
CC	GO:0044446	Intracellular organelle part	32/112	0,009	32	5	27
CC	GO:0044422	Organelle part	32/112	0,010	32	5	27
CC	GO:0044429	Mitochondrial part	8/112	0,018	8	0	8
CC	GO:0016469	Proton-transporting two-sector ATPase complex	5/112	0,022	5	0	5
CC	GO:0016592	Mediator complex	5/112	0,033	5	1	4
CC	GO:0043227	Membrane-bounded organelle	33/112	0,036	33	8	25
CC	GO:0044425	Membrane part	13/112	0,046	13	1	12
MF	GO:0003735	Structural constituent of ribosome	27/269	4,36E+01	27	0	27
MF	GO:0005198	Structural molecule activity	29/269	9,92E+03	29	1	28

### 3.2 Younger males versus older males

In the comparison between younger and older males, we did not find any significant differences in gene expression (Supplementary Table S3). Moreover, Gene Ontology and KEGG enrichment analyses did not identify any significantly enriched terms or pathways, respectively (Supplementary Tables S4, S5).

### 3.3 Younger females versus younger males

**FIGURE 4:**
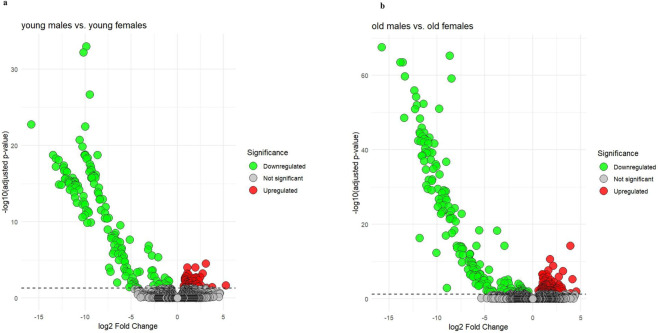
Volcano plots of differentially expressed genes with an adjusted p-value <0.05 between **(a)** younger males and younger females and **(b)** older males and older females. The dashed horizontal line corresponds to the threshold of significance.

**FIGURE 5:**
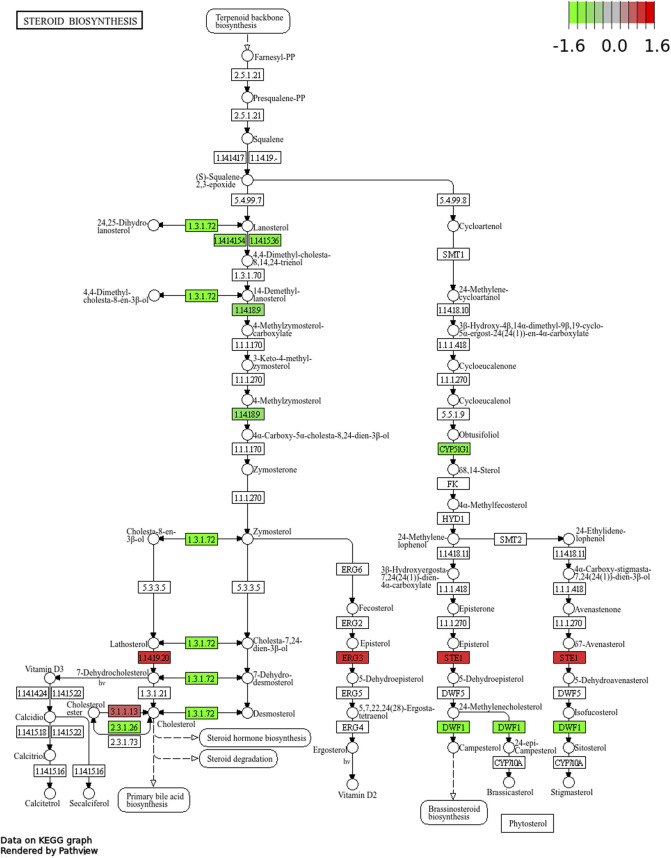
Visualization of the KEGG pathway for steroid biosynthesis. Differential gene expression data (log2 fold change) between younger females and younger males were mapped onto the pathway. Genes are represented as rectangles. Red rectangles indicate genes upregulated in younger males relative to younger females, whereas green rectangles indicate genes downregulated in younger males compared to younger females.

We identified 218 differentially expressed genes between younger females and younger males (Figure 4a). Gene Ontology enrichment analysis did not identify any significantly enriched terms (Supplementary Table S6). In contrast, KEGG enrichment analysis revealed steroid biosynthesis as the only significantly enriched pathway (Figure 1b). In particular, the largest differences emerged in genes participating in key steps of late cholesterol biosynthesis, namely, CYP51G1, ERG3, STE1, and DWF1 (Figure 5).

### 3.4 Older females versus older males

We found 359 differentially expressed genes between older females and older males (Figure 4b). Neither gene ontology nor KEGG Enrichment Analysis identified significantly enriched terms or pathways, respectively (Supplementary Tables S8, S9).

### 3.5 Shared and private genes in ageing

**FIGURE 6:**
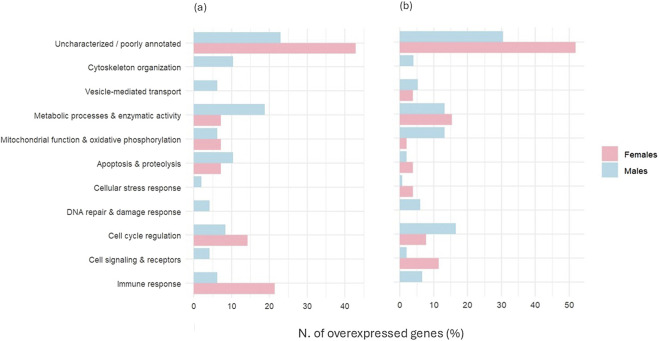
Bar plots showing sex-specific differences in gene expression patterns in younger **(a)** and older **(b)** shearwaters. Functional categories are displayed on the y-axis, while the x-axis represents the percentage of overexpressed genes within each sex. Colours indicate sex (females in pink, males in blue).

We found that 72% of the differentially expressed genes between sexes are shared between younger and older individuals (156 genes in total) and are therefore constitutively sex-biased throughout life. In contrast, 62 genes showed sex-specific differential expression only in younger individuals (Supplementary Table S9a), while 203 genes were differentially expressed exclusively in older individuals (Supplementary Table S9b). In younger individuals, the most pronounced sex-biased expression, based on log2 fold change, involved genes encoding proteins and factors involved in immune defense, metabolic processes, and cell cycle regulation (Figure 6a). In older individuals, sex-biased expression was most prominent in genes associated with mitochondrial function, cellular stress response, immunity, and DNA repair (Figure 6b).

## 4 Discussion

The results of our study show that within-sex ageing effects are weak, but between-sex differences are strong. The main within-sex differences were observed between younger and older females while the larger between-sex differences were observed in older individuals. Younger females showed lower expression of the gene NT5DC3, that encodes a protein belonging to the 5′-nucleotidase family, enzymes involved in nucleotide metabolism and cellular processes related to energy metabolism and cell proliferation. The higher expression of NT5DC3 in older females is consistent with findings of several experimental studies conducted on humans, pointing to this gene as a plausible molecular marker of ageing (Galamb et al., 2016; Meng et al., 2025).

We also found that the gene MMTAG2 (also known as C1orf35) was upregulated in younger females compared to older females. This gene encodes the multiple myeloma tumor-associated protein 2, which has RNA-binding activity. The biological function of MMTAG2 is not yet fully characterised, but some evidence suggests that this protein might play a role in the immune response in humans (Su et al., 2024). Although some studies found a decline in the expression of immune genes with age (see for wolves Charruau et al., 2016; for the giant panda Du et al., 2019), we do not know if downregulation of MMTAG2 in older shearwater females has any implications for their immunological ageing.

Gene Ontology and KEGG enrichment analyses indicated that, overall, younger females show an upregulation of genes involved in protein synthesis pathways (mitochondrial ribosomal genes) and oxidative phosphorylation. This is also consistent with previous findings on mice, where transcriptional changes in gene expression are downregulated with advancing age in dissipative networks (e.g., pathways producing ATP) and upregulated in stabilising networks (e.g., DNA repair, stress response, and inflammatory pathways), respectively (Brink et al., 2009). Furthermore, the reduction of metabolic activity with age is in line with observations of metabolic ageing at least in two bird species (Elliott et al., 2015; Rønning et al., 2014).

Comparison between younger females and younger males identified numerous differentially expressed genes and revealed steroid metabolism as the most significantly enriched pathway. Differentially expressed genes within this pathway were primarily involved in the late steps of cholesterol biosynthesis. Since cholesterol is the precursor of all steroid hormones, this pattern might reflect endocrine differences associated with reproductive physiology and sexual maturation (Bautista et al., 2013). Furthermore, during the early stages of reproductive life, male and female shearwaters differ in how they allocate resources between reproduction and somatic maintenance. Indeed, between 5 and 11 years of age, body mass increases more rapidly in males than in females, whereas females exhibit a greater increase in reproductive success between 5 and 9 years of age (Berardi et al., 2026a). Therefore, steroid hormones may play a central role in modulating these differences in resource allocation, as they are known to regulate life-history strategies (Hau et al., 2010), morphological development (Lindsay et al., 2016), and ovulation (Wadood et al., 2025), with testosterone and estradiol being key examples.

We also found that younger males have a significant higher expression of genes involved in DNA damage response, metabolic processes, and germline function (e.g., MDC1, PCCB, STAG3) than younger females. On the other hand, younger females show overexpressed genes that are primarily associated with immune pathway activation, mitochondrial function and cell cycle regulation (e.g., GBP1, LY6E, HMOX1). These results suggest that younger males invest more in metabolic efficiency, germline development, and genome maintenance, whereas younger females show a stronger investment in immune defense, mitochondrial function, and cellular stress response, together with active regulation of the cell cycle. Similar sex-biased patterns have been reported in other vertebrate studies (e.g., Hopkins et al., 2014; Klein and Flanagan, 2016).

Additionally, some of the genes overexpressed early in adulthood, including MDC1 (DNA damage checkpoint mediator), HMOX1 (oxidative stress response), and ACO1 (DNA repair protein), are also known biomarkers of ageing (Jung-Hee et al., 2015; Fernández-Mendívil et al., 2020). These genes could serve as suitable candidates for testing the antagonistic pleiotropy hypothesis (Williams, 1957; Mitteldorf, 2019), which posits that genes beneficial for fertility and other key aspects of fitness early in life may have detrimental effects at older ages.

In older shearwaters, gene expression patterns are partially consistent with those observed in younger individuals. Older males predominantly overexpress genes involved in mitochondrial function, immune response, DNA damage repair, and cell cycle regulation (e.g., Oxa1l, COX, ACO1, FANCE). In contrast, older females primarily overexpress genes associated with cellular stress, signaling, apoptosis, and enzymatic activity (e.g., FASLG, ATXN2, EGR1). These results suggest that males continue to invest in cellular maintenance and genomic stability, mainly through mechanisms of DNA damage prevention and repair, while also showing increased investment in immune defenses. Conversely, older females show increased activation of damage-management pathways and stress response mechanisms compared to younger females. A recent study on Scopoli’s shearwaters found that levels of age-related DNA damage and blood antioxidant enzymes did not differ between sexes (Berardi et al., 2026b). It may be that alternative strategies (damage prevention in males and damage management in females) converge on a similar functional outcome, i.e., maintaining genomic integrity which is fundamental to make life longer. For instance, species with longer lifespans, such as humans and naked mole-rats, are better in repairing DNA compared to short-lived species like mice (MacRae et al., 2015). Moreover, our findings are consistent with previous studies showing sex-specific ageing trajectories at the molecular level, with differences in mitochondrial function, immune regulation, and stress-response pathways (Moschinger et al., 2019; Zhu et al., 2024).

Overall, the transcriptomic profiles of males and females point to sustained investment in somatic maintenance into late life, supporting the long-standing view that wild birds exhibit relatively limited signs of ageing (Holmes et al., 2001). Contrary to what has been observed in other long-lived seabirds, females displayed a stronger ageing signature than males, as indicated by the downregulation of genes involved in mitochondrial function and the activation of stress-response pathways. In monogamous birds, where both partners share incubation and chick rearing, females incur the greatest reproductive costs due to egg production, which might contribute to their stronger ageing signature, as predicted by life-history theory (Bonduriansky et al., 2008). Indeed, at the time of sampling, females had just completed a month-long foraging trip at sea and had lost 15%–18% of their body mass, equivalent to the mass of the egg.

Consistent with this framework, females and males may rely on different reproductive strategies that lead to sex-specific optima for traits affecting longevity and ageing rate. During egg production, females are expected to tradeoff the transfer of key resources to the egg (e.g., antibodies, antioxidants) against investment in their own somatic maintenance (Hasselquist and Nilsson, 2009). In younger females, this tradeoff might be reflected in the strong expression of genes related to immune function and energy metabolism, whereas in later life the tradeoff would involve mainly genes associated with stress-response pathways.

Evidence across a wide range of species indicates that early-life investment in reproduction may entail physiological costs that manifest later in life (e.g., increased oxidative stress and cellular damage) thereby requiring the activation of compensatory mechanisms (Monaghan, 2008; Vágási et al., 2019). In contrast, reproductive costs in males appear to be more closely linked to competition for nest acquisition and defense, as well as extended fasting associated with the first incubation shift, which lasts 10–14 days (Becciu et al., 2012), potentially promoting a life-long strategy centered on damage prevention and cellular maintenance.

## 5 Conclusion

This is the first whole transcriptome study in a free-living bird to investigate candidate genes and pathways underlying sex-biased gene expression across age classes. We found molecular evidence for a sex-linked transcriptional signature, mainly associated with genes involved in immune function, stress response, energetics, and development. Age-related reductions in the expression of genes involved in energy metabolism were more pronounced in females than in males, while the greatest sex-specific differences occurred in immune and genome stability pathways. In males, transcriptional patterns remained relatively stable from early to late adulthood, with persistent overexpression of genes involved in DNA damage repair. By contrast, females exhibited a stronger focus on immune-related genes in early adulthood, which transitioned toward activation of stress-response pathways in late adulthood.

Beyond classical genetic theories of sex-specific ageing, our findings highlight that changes in gene expression also contribute to these differences, which we hypothesise are driven by the differentiation of sex-specific reproductive strategies during the egg-laying phase. Further analyses, including intermediate age classes and periods beyond egg-laying, will help improve our understanding of sex-biased molecular regulation and ageing processes in avian species. Finally, as this study focused on a single tissue, replication in other tissues is needed to provide a comprehensive view of ageing across the whole organism.

## Data Availability

The data presented in the study are deposited in the Zenodo repository, accession using the DOI 10.5281/zenodo.21263145.
