# Supplementary material for: Transcriptome analysis of ageing in a long-lived seabird reveals sex-specific differences across age classes in immune and DNA repair pathways
Source: Front Aging. 2026 Jul 15;7:1880546. doi: 10.3389/fragi.2026.1880546 (PMC13415676; doi:10.3389/fragi.2026.1880546)
Supplement: Supplementary file 2 [file DataSheet1.docx]

**Transcriptome analysis of ageing in a long-lived seabird reveals sex-specific differences across age classes in immune and DNA repair pathways**

**Beatrice Berardi^1,2^, Giacomo Dell’Omo^2^ and David Costantini^1^**

1Department of Ecological and Biological Sciences, University of Tuscia, Largo dell'Università s.n.c., 01100 Viterbo, Italy

2 *Ornis italica*, piazza Crati, 15, 00199 Rome, Italy

Table S1. Sample distribution by sex and age.

| Age | Females | Males |
| --- | --- | --- |
| 5 | 7 | 0 |
| 6 | 1 | 1 |
| 7 | 0 | 1 |
| 8 | 0 | 2 |
| 9 | 0 | 4 |
| 19 | 0 | 2 |
| 20 | 1 | 1 |
| 21 | 3 | 2 |
| 22 | 3 | 1 |
| 30 | 0 | 1 |
| 36 | 1 | 1 |
| Total | 16 | 16 |

Table S2. List of differentially expressed genes and their functions between younger females (yf) and older females (of). Log2FC = log2 fold change; Adj-p = adjusted p-value.

| **Gene ID** | **Higher expression in** | **log2FC** | **Adj-p** | **Gene name** | **Gene function** |
| --- | --- | --- | --- | --- | --- |
| ***1. Younger females vs older females*** | | | | | |
| 142075195 | yf | 2.86 | 0.003 | Unidentified | uncharacterized LOC142075195 |
| 142085436 | of | -3.88 | 0.026 | NT5DC3 | 5'-nucleotidase domain containing 3 |
| 142079685 | yf | 1.30 | 0.026 | MMTAG2 | chromosome 2 C1orf35 homolog, 2C transcript variant X2 |

**Tables S3 to S8 are provided in the Excel file “Results S3-S8.xlsx”, with one table per worksheet, because of their large size.**

Table S3. List of differentially expressed genes and their functions between younger and older males. Log2FC = log2 fold change; Adj-p = adjusted p-value.

Table S4. Results of Gene Ontology enrichment analysis of differentially expressed genes between younger and older males. The category refers to the functional classification of the gene. The Gene ratio represents the proportion of genes associated with a given function relative to the total number of genes in the input list, while the Bg ratio (background ratio) represents the proportion of genes associated with that function in the reference gene set. BP=Biological process, MF = Molecular function, CC= cellular component; Adj-p= adjusted p-value.

Table S5. Results of KEGG enrichment analysis of differentially expressed genes between younger and older males. KEGG ID refers to specific pathways or genes in the KEGG database. The Gene ratio represents the proportion of genes associated with a given function relative to the total number of genes in the input list, while the Bg ratio (background ratio) represents the proportion of genes associated with that function in the reference gene set. Adj-p= adjusted p-value.

Table S6. Results of Gene Ontology enrichment analysis of differentially expressed genes between younger females and younger males. The category refers to the functional classification of the gene. The Gene ratio represents the proportion of genes associated with a given function relative to the total number of genes in the input list, while the Bg ratio (background ratio) represents the proportion of genes associated with that function in the reference gene set. BP=Biological process, MF = Molecular function, CC= cellular component; Adj-p= adjusted p-value.

Table S7. Results of Gene Ontology enrichment analysis of differentially expressed genes between older females and older males. The category refers to the functional classification of the gene. The Gene ratio represents the proportion of genes associated with a given function relative to the total number of genes in the input list, while the Bg ratio (background ratio) represents the proportion of genes associated with that function in the reference gene set. BP=Biological process, MF = Molecular function, CC= cellular component; Adj-p= adjusted p-value.

Table S8. Results of KEGG enrichment analysis of differentially expressed genes between older females and older males. KEGG ID refers to specific pathways or genes in the KEGG database. The Gene ratio represents the proportion of genes associated with a given function relative to the total number of genes in the input list, while the Bg ratio (background ratio) represents the proportion of genes associated with that function in the reference gene set. Adj-p= adjusted p-value.

Table S9. List of genes and their functions that are sex-specifically expressed exclusively in the younger age group (a) and in the older age group (b), ordered by decreasing log2 fold change (log2FC). Adj-p = adjusted p-value; yf = younger females; ym = younger males; of = older females; om = older males. Genes shown in bold are those for which direct or indirect associations with ageing processes have been reported.

| *(a) Younger females versus younger males* | | | | | | | | |
| --- | --- | --- | --- | --- | --- | --- | --- | --- |
| ID | **Higher expression in** | **log2FC** | **Adj_p** | **Gene name** | **Gene function** | |  | |
| 1 | ym | 5.28 | 0.020 | Unidentified | Secreted lipocalin; small hydrophobic molecule binding | | |  |
| 2 | ym | 3.79 | 0.036 | H2B-VIII | Core histone protein; chromatin structure and gene regulation | | |  |
| 3 | ym | 3.18 | 0.044 | FCGBP | IgG Fc-binding mucosal immune protein | | |  |
| 4 | ym | 2.55 | 0.020 | Unidentified | Unknown function |  | |  |
| 5 | ym | 2.33 | 0.029 | EFR3B | Plasma membrane protein regulating phosphoinositide synthesis | | |  |
| 6 | ym | 2.32 | 0.005 | ACR | Serine protease involved in fertilization | | |  |
| 7 | ym | 2.25 | 0.034 | Tmem79 | Transmembrane protein involved in skin barrier function | | |  |
| 8 | ym | 2.14 | 0.014 | MYO18A | Actin-associated motor protein; cytoskeletal organization | | |  |
| 9 | ym | 2.04 | 0.002 | DNMBP | Endocytic adaptor protein interacting with dynamin | | |  |
| 10 | ym | 2.02 | 0.013 | Galt | Galactose metabolism enzyme (Leloir pathway) | | |  |
| 11 | ym | 2.00 | 0.002 | ACR | Serine protease involved in fertilization | | |  |
| 12 | ym | 1.96 | <0.001 | STAG3 | Meiotic cohesin complex component | | |  |
| 13 | ym | 1.81 | 0.023 | ACR | Serine protease involved in fertilization | | |  |
| 14 | ym | 1.79 | 0.002 | Taf1c | RNA polymerase I transcription factor subunit | | |  |
| 15 | ym | 1.76 | 0.006 | PIGO | GPI-anchor biosynthesis enzyme | | |  |
| 16 | ym | 1.75 | 0.014 | ACTL7A | Actin-related protein; cytoskeletal organization | | |  |
| 17 | **ym** | **1.74** | **0.004** | **ABCA2** | **Lipid transporter (ABC transporter family)** | | |  |
| 18 | ym | 1.70 | 0.020 | Atosb | Poorly characterized protein; unknown function | | |  |
| 19 | ym | 1.70 | 0.037 | Unidentified | Unknown function |  | |  |
| 20 | ym | 1.66 | 0.035 | RND2 | Small GTPase; cytoskeleton and signaling regulation | | |  |
| 21 | **ym** | **1.60** | **0.045** | **MDC1** | **DNA damage checkpoint mediator** | | |  |
| 22 | **ym** | **1.59** | **0.020** | **Huwe1** | **E3 ubiquitin ligase; protein degradation** | | |  |
| 23 | ym | 1.49 | 0.006 | SUSD6 | Extracellular sushi domain-containing protein | | |  |
| 24 | ym | 1.47 | 0.042 | DCTN3 | Dynactin complex subunit; microtubule-based transport | | |  |
| 25 | ym | 1.43 | 0.011 | Frs3 | Adaptor protein in growth factor signaling | | |  |
| 26 | ym | 1.40 | 0.035 | Nphp4 | Ciliary protein; centrosome function | | |  |
| 27 | ym | 1.39 | 0.011 | OTOL1 | Extracellular matrix protein; inner ear structure | | |  |
| 28 | ym | 1.33 | 0.007 | STYXL1 | Regulatory adaptor protein; signaling pathways | | |  |
| 29 | **ym** | **1.33** | **0.034** | **ARSK** | **Lysosomal pathway** |  | |  |
| 30 | **ym** | **1.29** | **0.042** | **GRSF1** | **RNA-binding protein; mitochondrial RNA regulation** | | |  |
| 31 | ym | 1.28 | 0.020 | Auts2 | Poorly characterized secreted protein | | |  |
| 32 | ym | 1.25 | 0.011 | Xylb | Carbohydrate metabolism enzyme | | |  |
| 33 | ym | 1.20 | 0.030 | FBXO4 | F-box protein; ubiquitin ligase complex component | | |  |
| 34 | ym | 1.19 | <0.001 | Chst5 | Carbohydrate sulfotransferase; glycosaminoglycan modification | | |  |
| 35 | ym | 1.16 | 0.009 | TBC1D2 | Rab GTPase-activating protein; vesicle trafficking | | |  |
| 36 | ym | 1.14 | 0.050 | fam162b | Mitochondrial apoptosis-related protein | | |  |
| 37 | ym | 1.08 | 0.030 | Rcl1 | RNA processing enzyme | | |  |
| 38 | ym | 1.07 | <0.001 | FAM219A | Poorly characterized protein; unknown function | | |  |
| 39 | **ym** | **1.05** | **0.019** | **USP28** | **Deubiquitinating enzyme** | | |  |
| 40 | ym | 1.00 | 0.013 | Unidentified | Unknown function |  | |  |
| 41 | ym | 0.94 | 0.007 | Tmed7 | ER-Golgi trafficking protein | | |  |
| 42 | ym | 0.90 | 0.036 | PCCB | Mitochondrial enzyme in fatty acid metabolism | | |  |
| 43 | ym | 0.89 | 0.016 | SHLD1 | DNA repair factor (double-strand break repair) | | |  |
| 44 | **ym** | **0.88** | **0.001** | **SSBP2** | **Single-stranded DNA-binding protein; genome stability** | | |  |
| 45 | **ym** | **0.87** | **0.039** | **SETD6** | **Histone lysine methyltransferase; epigenetic regulation** | | |  |
| 46 | ym | 0.86 | 0.005 | TPCN1 | Endolysosomal ion channel | | |  |
| 47 | ym | 0.80 | 0.031 | FNTA | Protein prenylation enzyme | | |  |
| 48 | **ym** | **0.69** | **0.023** | **Metap1d** | **Mitochondrial methionine aminopeptidase** | | |  |
| 49 | **yf** | **-4.99** | **0.006** | **CENPH** | **Centromere-associated protein; chromosome segregation** | | |  |
| 50 | yf | -4.50 | 0.038 | Unidentified | Unknown function |  | |  |
| 51 | yf | -4.40 | 0.042 | SMARCA2 | Chromatin remodeling complex ATPase | | |  |
| 52 | **yf** | **-4.25** | **0.048** | **CCNB1** | **Cellular cycle** |  | |  |
| 53 | **yf** | **-3.11** | **<0.001** | **LY6E** | **Cell surface immune-related protein** | | |  |
| 54 | **yf** | **-2.67** | **<0.001** | **GBP1** | **Interferon-inducible GTPase; immune response** | | |  |
| 55 | yf | -2.60 | 0.020 | Cracr2a | Calcium channel regulator (store-operated calcium entry) | | |  |
| 56 | **yf** | **-2.49** | **0.001** | **HMOX1** | **Heme degradation enzyme; oxidative stress response** | | |  |
| 57 | yf | -2.24 | 0.005 | Unidentified | Unknown function |  | |  |
| 58 | **yf** | **-2.20** | **0.040** | **FADS2** | **Polyunsaturated fatty acid metabolism** |  | |  |
| 59 | **yf** | **-2.06** | **<0.001** | **EFHD1** | **Calcium-binding protein; cytoskeletal regulation** | | |  |
| 60 | **yf** | **-1.43** | **0.013** | **SOCS4** | **Negative regulator of cytokine signaling** | | |  |
| 61 | yf | -0.97 | 0.023 | Unidentified | Unknown function |  | |  |
| 62 | **yf** | **-0.89** | **0.006** | **MTERF3** | **Mitochondrial transcription termination factor** | | |  |

| *(b) Older females versus older males* | | | | | |
| --- | --- | --- | --- | --- | --- |
| N | **Higher expression in** | **log2FC** | **Adj_p** | **Gene name** | **Gene function** |
| 1 | om | 4.54 | 0.010 | TMEM117 | Apoptosis and membrane organization |
| 2 | om | 4.14 | <0.001 | psuG | Unknown function |
| 3 | om | 3.96 | 0.030 | DCX | Microtubule-associated protein involved in neuronal migration |
| 4 | om | 3.15 | <0.001 | Unidentified | Unknown function |
| 5 | om | 3.14 | 0.008 | Unidentified | Unknown function |
| 6 | om | 3.06 | 0.046 | Unidentified | Structural component of small ribosomal subunit |
| 7 | om | 3.00 | 0.002 | AMACR | Peroxisomal and mitochondrial lipid metabolism enzyme |
| 8 | om | 2.99 | <0.001 | MANSC4 | Predicted secreted or extracellular protein |
| 9 | om | 2.70 | <0.001 | HOXD10 | Homeobox transcription factor involved in developmental patterning |
| 10 | om | 2.70 | <0.001 | LYVE1 | Lymphatic endothelial receptor involved in hyaluronan binding and cell adhesion |
| 11 | om | 2.63 | 0.005 | Unidentified | Unknown function |
| 12 | om | 2.61 | 0.011 | OR52E8 | Unknown function |
| 13 | om | 2.50 | 0.001 | Unidentified | Unknown function |
| 14 | om | 2.50 | 0.042 | Unidentified | Unknown function |
| 15 | om | 2.37 | 0.050 | Unidentified | Structural component of small ribosomal subunit |
| 16 | om | 2.36 | <0.001 | Blvrb | Redox enzyme involved in heme metabolism |
| 17 | om | 2.36 | 0.036 | Klhdc8b | Kelch domain-containing protein; likely involved in protein-protein interactions |
| 18 | om | 2.35 | 0.002 | ZBTB16 | Transcriptional regulator with zinc finger and BTB domains |
| 19 | om | 2.34 | 0.046 | Unidentified | Unknown function |
| 20 | om | 2.31 | 0.001 | Susd3 | Extracellular matrix protein with sushi domains; cell adhesion |
| 21 | om | 2.26 | 0.033 | SPR | Enzyme involved in tetrahydrobiopterin biosynthesis |
| 22 | om | 2.23 | 0.003 | ADGRV1 | Adhesion G protein-coupled receptor involved in signaling |
| 23 | om | 2.22 | 0.002 | Unidentified | Unknown function |
| 24 | om | 2.20 | 0.041 | FREM1 | Extracellular matrix protein involved in tissue integrity |
| 25 | om | 2.18 | <0.001 | TRIM27 | E3 ubiquitin ligase involved in protein degradation |
| 26 | om | 2.18 | 0.011 | smc2 | Chromosome cohesion and condensation protein |
| 27 | om | 2.14 | 0.003 | PET100 | Mitochondrial protein required for cytochrome c oxidase assembly |
| 28 | om | 2.13 | <0.001 | ptcd2 | RNA-binding protein with pentatricopeptide repeats |
| 29 | om | 2.13 | 0.027 | Unidentified | Enzyme involved in arylamine acetylation |
| 30 | **om** | **2.07** | **0.001** | **UBE2L5** | **Ubiquitin-conjugating enzyme involved in protein degradation** |
| 31 | **om** | **2.06** | **0.010** | **POLD2** | **DNA replication accessory subunit** |
| 32 | om | 2.01 | 0.004 | WDR83 | WD repeat scaffold protein; protein-protein interactions |
| 33 | om | 2.00 | 0.015 | Ppif | Mitochondrial peptidyl-prolyl isomerase |
| 34 | om | 1.99 | 0.045 | Shank3 | Cytoskeletal adaptor protein |
| 35 | om | 1.96 | 0.004 | LAMTOR4 | Endolysosomal adaptor regulating MAPK and mTOR signaling |
| 36 | om | 1.96 | 0.007 | TMOD4 | Actin filament capping protein |
| 37 | **om** | **1.93** | **0.005** | **NDUFS8** | **Mitochondrial complex I subunit** |
| 38 | om | 1.93 | 0.025 | Dnajb5 | Molecular chaperone of Hsp40 family |
| 39 | om | 1.91 | <0.001 | Unidentified | Transcription factor involved in erythroid differentiation |
| 40 | om | 1.91 | 0.036 | CP37 | Anti-inflammatory phospholipid-binding protein |
| 41 | om | 1.90 | <0.001 | HEXB | Lysosomal enzyme involved in glycosaminoglycan degradation |
| 42 | om | 1.90 | 0.005 | SGTB | Co-chaperone protein with TPR domains |
| 43 | om | 1.90 | 0.049 | Unidentified | Zinc finger transcription factor |
| 44 | om | 1.89 | 0.045 | FAM237A | Poorly characterized protein; unknown function |
| 45 | **om** | **1.87** | **0.022** | **FANCE** | **DNA repair protein (Fanconi anemia pathway)** |
| 46 | om | 1.83 | <0.001 | Unidentified | Unknown function |
| 47 | **om** | **1.82** | **0.029** | **ATG9A** | **Autophagy-related membrane trafficking protein** |
| 48 | **om** | **1.82** | **0.031** | **CTSL** | **Lysosomal cysteine protease** |
| 49 | om | 1.81 | 0.004 | Unidentified | Mitochondrial complex I subunit |
| 50 | om | 1.81 | 0.006 | C19orf53 | Poorly characterized protein; unknown function |
| 51 | om | 1.80 | <0.001 | NSA2 | Ribosome biogenesis factor |
| 52 | **om** | **1.79** | **0.004** | **TIMM10** | **Mitochondrial inner membrane translocase** |
| 53 | om | 1.79 | 0.020 | RUSF1 | Poorly characterized intracellular protein |
| 54 | om | 1.77 | 0.045 | PPT2 | Lysosomal depalmitoylation enzyme |
| 55 | om | 1.76 | 0.001 | LRP2BP | Adaptor protein interacting with LRP2 receptor |
| 56 | om | 1.75 | 0.040 | Unidentified | Unknown function |
| 57 | om | 1.74 | 0.007 | SLC12A2 | Na-K-Cl cotransporter; ion transport |
| 58 | **om** | **1.71** | **<0.001** | **RPS6** | **Ribosomal protein of small subunit; translation regulation** |
| 59 | om | 1.71 | 0.003 | POLR3GL | RNA polymerase III subunit |
| 60 | **om** | **1.71** | **0.017** | **Oxa1l** | **Mitochondrial inner membrane protein insertase** |
| 61 | om | 1.70 | <0.001 | COMMD7 | Protein involved in NF-kB signaling regulation |
| 62 | om | 1.70 | 0.017 | NABP2 | Single-stranded nucleic acid-binding protein |
| 63 | **om** | **1.66** | **0.001** | **mrpl52** | **Mitochondrial ribosomal protein** |
| 64 | om | 1.66 | 0.029 | Unidentified | Unknown function |
| 65 | om | 1.66 | 0.044 | Unidentified | Unknown function |
| 66 | om | 1.65 | 0.041 | Unidentified | Unknown function |
| 67 | **om** | **1.63** | **0.008** | **RPS23** | **Ribosomal protein of small subunit** |
| 68 | om | 1.61 | 0.044 | castor2 | mTORC1 arginine sensor component |
| 69 | om | 1.60 | 0.018 | GCAT | Mitochondrial glycine metabolism enzyme |
| 70 | om | 1.60 | 0.024 | ETFBKMT | Mitochondrial protein methyltransferase |
| 71 | om | 1.58 | <0.001 | SNX31 | Endosomal trafficking protein |
| 72 | om | 1.53 | 0.014 | Nagk | Enzyme of amino sugar metabolism |
| 73 | **om** | **1.53** | **0.031** | **MRPL50** | **Mitochondrial ribosomal protein** |
| 74 | om | 1.52 | <0.001 | MOCS2 | Molybdenum cofactor biosynthesis enzyme |
| 75 | om | 1.52 | 0.004 | GNG12 | G protein gamma subunit; signal transduction |
| 76 | om | 1.51 | <0.001 | CLTA | Clathrin-mediated endocytosis component |
| 77 | om | 1.51 | 0.032 | fbl | Nucleolar rRNA methyltransferase |
| 78 | om | 1.50 | <0.001 | TTBK1 | Microtubule-associated kinase |
| 79 | om | 1.49 | 0.048 | COPS6 | Protein complex regulating ubiquitin ligases |
| 80 | om | 1.45 | <0.001 | SLC1A4 | Neutral amino acid transporter |
| 81 | om | 1.45 | 0.007 | INIP | DNA damage response adaptor protein |
| 82 | **om** | **1.45** | **0.028** | **COX7C** | **Cytochrome c oxidase subunit; mitochondrial respiration** |
| 83 | **om** | **1.44** | **0.020** | **ISCA1** | **Iron-sulfur cluster assembly protein** |
| 84 | om | 1.44 | 0.044 | MALT1 | Scaffold protein involved in NF-kB activation |
| 85 | om | 1.43 | 0.004 | Psat1 | Serine biosynthesis enzyme |
| 86 | om | 1.41 | 0.020 | Elob | Elongin complex component; transcription and ubiquitination |
| 87 | **om** | **1.40** | **0.027** | **Atp5me** | **ATP synthase membrane subunit** |
| 88 | om | 1.39 | <0.001 | RFX3 | Transcription factor regulating ciliogenesis |
| 89 | om | 1.36 | <0.001 | MOCS2 | Molybdenum cofactor biosynthesis protein |
| 90 | om | 1.36 | 0.019 | CDC42SE2 | CDC42 effector protein; actin regulation |
| 91 | **om** | **1.36** | **0.026** | **RPL37** | **Ribosomal protein of large subunit** |
| 92 | om | 1.35 | 0.005 | SLC25A15 | Mitochondrial ornithine transporter |
| 93 | om | 1.34 | 0.027 | Unidentified | Unknown function |
| 94 | om | 1.33 | 0.033 | SUB1 | Transcriptional coactivator |
| 95 | **om** | **1.32** | **0.015** | **cdk4** | **Cell cycle kinase (G1 phase progression)** |
| 96 | om | 1.30 | <0.001 | Unidentified | Unknown function |
| 97 | om | 1.30 | <0.001 | NUDT2 | Nudix hydrolase; nucleotide metabolism |
| 98 | om | 1.30 | 0.012 | ATG10 | Autophagy-related ubiquitin-like protein |
| 99 | om | 1.29 | <0.001 | ZDHHC21 | Palmitoyltransferase; protein lipid modification |
| 100 | om | 1.29 | 0.032 | Arhgef39 | Rho guanine nucleotide exchange factor |
| 101 | om | 1.26 | <0.001 | RIMOC1 | Endosomal trafficking regulator |
| 102 | om | 1.26 | 0.046 | ANO10 | Calcium-activated chloride channel |
| 103 | om | 1.25 | 0.048 | Unidentified | Oxidoreductase enzyme |
| 104 | om | 1.23 | 0.003 | HSDL2 | TCA cycle enzyme subunit |
| 105 | **om** | **1.23** | **0.045** | **IDH3G** | **Mitochondrial complex assembly factor** |
| 106 | **om** | **1.21** | **0.015** | **LYRM7** | **Mitochondrial iron homeostasis protein** |
| 107 | **om** | **1.19** | **0.003** | **FXN** | **Heme transporter** |
| 108 | om | 1.18 | 0.045 | slc49a3 | Vesicle trafficking complex component |
| 109 | om | 1.17 | 0.011 | TRAPPC13 | GPI-anchor biosynthesis enzyme |
| 110 | om | 1.17 | 0.014 | PIGG | Prostaglandin receptor; inflammatory signaling |
| 111 | om | 1.16 | 0.038 | PTGER4 | Unknown function |
| 112 | om | 1.16 | 0.043 | Unidentified | Cytoskeletal membrane-associated protein |
| 113 | om | 1.15 | 0.007 | EPB42 | Aminopeptidase enzyme |
| 114 | om | 1.14 | <0.001 | LNPEP | Mitochondrial ribosomal protein |
| 115 | om | 1.13 | <0.001 | MRPS30 | DNA repair enzyme (base excision repair) |
| 116 | om | 1.12 | <0.001 | QNG1 | General transcription factor |
| 117 | om | 1.10 | <0.001 | Btf3 | RNA splicing and snRNP assembly factor |
| 118 | om | 1.10 | <0.001 | Smn1 | Unknown function |
| 119 | om | 1.10 | 0.007 | Unidentified | DNA damage recognition and nucleotide excision repair protein |
| 120 | **om** | **1.09** | **0.002** | **XPA** | **E3 ubiquitin ligase** |
| 121 | om | 1.08 | 0.010 | Kcmf1 | RNA-binding zinc finger protein |
| 122 | om | 1.08 | 0.023 | ZCCHC7 | Adaptor protein complex subunit; vesicle trafficking |
| 123 | om | 1.07 | <0.001 | AP3S1 | Linker histone; chromatin compaction |
| 124 | om | 1.07 | 0.049 | Unidentified | TCA cycle enzyme; iron metabolism |
| 125 | **om** | **1.06** | **<0.001** | **ACO1** | **DNA repair protein (nucleotide excision repair)** |
| 126 | **om** | **1.06** | **0.004** | **RAD23B** | **mRNA surveillance and ribosome rescue factor** |
| 127 | om | 1.03 | 0.002 | PELO | Unknown function |
| 128 | om | 1.02 | 0.008 | Unidentified | Aminoacyl-tRNA synthetase |
| 129 | om | 1.01 | 0.001 | TARS1 | Transmembrane protein; unknown function |
| 130 | om | 1.01 | 0.001 | TMEM171 | Lysosomal cysteine protease |
| 131 | om | 1.01 | 0.008 | CTSC | Protein prenylation enzyme subunit |
| 132 | om | 1.00 | 0.019 | PGGT1B | Poorly characterized protein |
| 133 | om | 0.99 | 0.001 | DMXL2 | SNARE protein; vesicle fusion |
| 134 | om | 0.98 | 0.032 | VAMP8 | DNA damage checkpoint exonuclease |
| 135 | **om** | **0.97** | **<0.001** | **RAD1** | **Cell cycle kinase regulating transcription** |
| 136 | **om** | **0.96** | **0.001** | **CDK7** | **DNA damage-binding protein** |
| 137 | **om** | **0.96** | **0.039** | **DDB2** | **Glycosyltransferase; glycoprotein biosynthesis** |
| 138 | om | 0.93 | 0.002 | B4GALT1 | Tubulin folding chaperone |
| 139 | om | 0.93 | 0.003 | TBCA | Sialyltransferase; glycosylation enzyme |
| 140 | om | 0.90 | <0.001 | ST6GAL1 | Ankyrin repeat scaffolding protein |
| 141 | om | 0.90 | 0.010 | ANKRA2 | tRNA methyltransferase |
| 142 | om | 0.88 | 0.009 | TRMT10B | Small secreted protein; poorly characterized |
| 143 | om | 0.86 | 0.020 | Unidentified | Lysosomal glycosidase |
| 144 | om | 0.84 | 0.020 | FUCA2 | Translation regulatory protein |
| 145 | om | 0.83 | 0.002 | Paip1 | Aldehyde metabolism enzyme |
| 146 | **om** | **0.83** | **0.014** | **ALDH7A1** | **E3 ubiquitin ligase with PHD/RING domains** |
| 147 | om | 0.78 | 0.007 | Uhrf2 | Unknown function |
| 148 | om | 0.78 | 0.035 | Unidentified | RNA polymerase I subunit |
| 149 | om | 0.76 | 0.033 | POLR1E | Nudix hydrolase; mitochondrial metabolism |
| 150 | om | 0.72 | 0.008 | Nudt12 | DNA replication initiation factor |
| 151 | om | 0.59 | 0.005 | ORC3 | Unknown function |
| 152 | of | -8.95 | 0.001 | Unidentified | Transmembrane protein; unknown function |
| 153 | of | -5.96 | <0.001 | TMEM8B | Unknown function |
| 154 | of | -5.68 | <0.001 | Unidentified | Unknown function |
| 155 | of | -5.16 | <0.001 | Unidentified | G protein alpha subunit; signal transduction |
| 156 | of | -5.09 | <0.001 | GNAQ | Unknown function |
| 157 | of | -4.63 | 0.012 | Unidentified | Unknown function |
| 158 | of | -4.53 | <0.001 | Unidentified | Unknown function |
| 159 | of | -4.17 | 0.006 | Unidentified | Small nucleolar RNA; rRNA modification |
| 160 | of | -4.13 | 0.013 | Unidentified | Cell adhesion molecule |
| 161 | of | -3.40 | 0.012 | Mcam | Fatty acid desaturation enzyme |
| 162 | of | -3.29 | 0.001 | Unidentified | Transfer RNA for cysteine |
| 163 | of | -3.00 | 0.003 | Unidentified | Monooxygenase-like oxidoreductase |
| 164 | of | -2.96 | <0.001 | Moxd2 | Unknown function |
| 165 | of | -2.71 | 0.019 | H2-K1 | Unknown function |
| 166 | of | -2.61 | <0.001 | Unidentified | Tetraspanin; cell adhesion and signaling |
| 167 | of | -2.56 | 0.009 | Cd82 | RhoGEF protein; vesicle trafficking |
| 168 | of | -2.30 | 0.009 | PSD3 | Apoptosis-inducing ligand (FasL) |
| 169 | **of** | **-2.27** | **0.037** | **FASLG** | **Neuronal signaling adaptor protein** |
| 170 | of | -2.26 | 0.001 | PACSIN2 | Unknown function |
| 171 | of | -2.25 | 0.044 | Unidentified | Rab GTPase-activating protein |
| 172 | of | -2.07 | 0.008 | TBC1D30 | RhoGAP involved in axon guidance |
| 173 | of | -2.00 | 0.017 | Srgap1 | Small GTPase family member |
| 174 | of | -1.77 | 0.039 | Rasd1 | Plasma membrane calcium pump |
| 175 | of | -1.74 | 0.032 | ATP2B1 | Epoxide metabolism enzyme |
| 176 | **of** | **-1.73** | **0.032** | **EPHX2** | **Homeobox transcription factor** |
| 177 | of | -1.67 | 0.036 | BSX | Inositol polyphosphate phosphatase |
| 178 | **of** | **-1.58** | **0.013** | **MINPP1** | **Stress-activated protein kinase** |
| 179 | **of** | **-1.58** | **0.032** | **MAPKAPK2** | **Ras guanine nucleotide exchange factor** |
| 180 | of | -1.57 | 0.014 | RASGEF1C | Phospholipid translocation protein |
| 181 | of | -1.51 | 0.021 | Plscr1 | Transcriptional regulator; Wnt signaling modulator |
| 182 | of | -1.48 | 0.011 | CXXC5 | Unknown function |
| 183 | of | -1.47 | 0.003 | AKAP8L | E3 ubiquitin ligase (TRIM family) |
| 184 | of | -1.39 | 0.007 | Trim8 | Small G protein signaling modulator |
| 185 | of | -1.38 | 0.010 | SGSM2 | Unknown function |
| 186 | of | -1.31 | <0.001 | Unidentified | Rho GTPase-activating protein |
| 187 | of | -1.27 | 0.022 | Arhgap39 | Calcineurin signaling regulator |
| 188 | of | -1.21 | 0.006 | RCAN1 | Unknown function |
| 189 | of | -1.14 | 0.004 | Unidentified | Immediate early transcription factor |
| 190 | **of** | **-1.12** | **0.008** | **EGR1** | **Glucose transporter (GLUT1)** |
| 191 | of | -1.11 | 0.002 | SLC2A1 | RNA-binding protein involved in stress granules |
| 192 | **of** | **-1.10** | **0.027** | **ATXN2** | **Extracellular serine protease** |
| 193 | of | -1.04 | 0.041 | PRSS12 | Poorly characterized protein |
| 194 | of | -0.94 | 0.024 | Unidentified | G protein-coupled receptor |
| 195 | of | -0.94 | 0.042 | GPR174 | Glycolysis regulatory enzyme |
| 196 | **of** | **-0.93** | **0.027** | **PFKFB2** | **Axonemal dynein motor protein** |
| 197 | of | -0.92 | 0.035 | DNAH17 | Unknown function |
| 198 | of | -0.89 | 0.013 | SPATA2 | Unknown function |
| 199 | of | -0.89 | 0.038 | Unidentified | Unknown function |
| 200 | of | -0.86 | 0.007 | Unidentified | Molybdenum cofactor biosynthesis enzyme |
| 201 | of | -0.73 | 0.040 | MOCS1 | Mitochondrial RNA-binding protein |
| 202 | of | -0.71 | 0.020 | ptcd3 | Sphingomyelin biosynthesis enzyme |
| 203 | **of** | **-0.47** | **0.011** | **SGMS1** | **Lipid metabolism** |
